# Early Warnings of Regime Shift When the Ecosystem Structure Is Unknown

**DOI:** 10.1371/journal.pone.0045586

**Published:** 2012-09-21

**Authors:** William A. Brock, Stephen R. Carpenter

**Affiliations:** 1 Department of Economics, University of Wisconsin, Madison, Wisconsin, United States of America; 2 Center for Limnology, University of Wisconsin, Madison, Wisconsin, United States of America; Universitat Pompeu Fabra, Spain

## Abstract

Abrupt changes in dynamics of an ecosystem can sometimes be detected using monitoring data. Using nonparametric methods that assume minimal knowledge of the underlying structure, we compute separate estimates of the drift (deterministic) and diffusion (stochastic) components of a general dynamical process, as well as an indicator of the conditional variance. Theory and simulations show that nonparametric conditional variance rises prior to critical transition. Nonparametric diffusion rises also, in cases where the true diffusion function involves a critical transition (sometimes called a noise-induced transition). Thus it is possible to discriminate noise-induced transitions from other kinds of critical transitions by comparing time series for the conditional variance and the diffusion function. Monte Carlo analysis shows that the indicators generally increase prior to the transition, but uncertainties of the indicators become large as the ecosystem approaches the transition point.

## Introduction

Regime shifts are massive changes in ecosystem structure and feedbacks that sometimes occur with little warning [1]–[3]. Examples include degradation of rangelands and forests, loss of fish stocks, and eutrophication of lakes and reservoirs. Massive changes that are slow to reverse can cause significant losses that affect human wellbeing [4]. Therefore regime shifts have attracted attention from ecosystem managers as well as researchers.

Regime shifts often come as surprises. In certain situations, however, statistical early warning signals can be measured in advance of regime shifts [3], [5]–[7]. These statistical indicators include changes in the autocorrelation and variance of time series. An expanding literature evaluates the situations where early warnings may or may not occur, as well as the empirical evidence for early warning indicators. Various indicators have successfully provided early warnings in applications to paleoclimate time series [8], [9], lab experiments on plankton [10], [11], and a whole-lake food web experiment [12]. Thus ecosystems do exhibit early warning indicators for cases of practical interest. In at least some cases, warnings could decrease the incidence of surprising regime shifts. Nonetheless they cannot completely eliminate surprise. For example, if ecosystems are forced rapidly into degraded states, environmental shocks are too large, or observations are too imprecise then there will be no early warning [13], [14].

A fundamental problem of early warning indicators is that the true process that generates the observed data is not known. If we knew in advance the key variables that control a critical transition, we would simply measure those variables. Sometimes a plausible model for nonlinear transitions can be fitted with acceptably small errors [15], [16]. However, the data must include several instances of the regime shift and adequate data sets are rare in ecology [2].

Nonparametric regression, in contrast, requires relatively few assumptions about the true data-generating process. Here we investigate the use of nonparametric regression to estimate key features of the underlying stochastic dynamic process and provide early warnings of impending critical transition. This paper provides substantial information not found in previous papers about early warnings using nonparametrics [17], [18]. We introduce a conditional variance estimator that, in theory, should perform better than statistics used in earlier papers. While Dakos et al. (2012) explain how to compute nonparametrics, this paper provides a thorough explanation of underlying theory and why it works.

## Methods

This section first introduces a general model for nonlinear stochastic time series that may contain regime shifts, and indicators that may serve as early warnings. We then explain how the indicators can be computed from observed time series using nonparametric methods. In order to illustrate and test the indicators, we conducted simulation experiments using different versions of a lake eutrophication model, as well as a more complex lake food web model. The rationale and procedures for the simulation experiments are explained.

### A General Model

In many cases it may be reasonable to assume that observed ecosystem dynamics are generated by a dynamical stochastic process of the general form
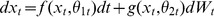
(1)


According to [1], the one-step rate of change in the time series of x (the state variable, which is possibly multi-dimensional) depends on a deterministic component f() and a stochastic component g(). It is conventional to refer to f() as the drift function and g() as the diffusion function. Either f() or g(), or both, may be nonlinear functions that are subject to a critical transition. Thus the model can represent most of the critical transitions that are commonly studied in ecology, depending on the specific form of f() and g(). The critical transition, if it occurs, happens when the variables θ_1t_ or θ_2t_ cross a critical threshold. An early warning, if it occurs, is a statistic that changes discernibly as either θ_1t_ or θ_2t_ approach the critical threshold. Changes in θ_1t_ and θ_2t_ are assumed to be slow relative to the rate of change in x. In [1], {W_t_} is a standardized Wiener process.

In order to generalize the methods presented below, we assume that the state vector is not directly observed, but instead we observe a variable
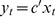
(2)


Usually y is lower-dimensioned than x and c is a matrix that converts x to y (c’ denotes transpose). Thus equation [2] can represent cases where only a few dimensions are sampled from a high-dimensional system.

Our earlier work pointed out that 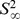, the long-run stationary variance of x for the linearization of [1] around a deterministic steady state, becomes infinite at a critical point caused by a generic bifurcation where the leading eigenvalue(s) approach the boundary of the stable region [6], [19]. Here we attempt to go beyond linearizations by using nonparametric estimators of general functions. This approach avoids restrictions to particular functional forms and also allows one to help separate critical transitions caused by bifurcations in the drift from critical transitions caused by increases in the diffusion function, called “noise induced transitions” [20], [21]. Such transitions encompass a wide spectrum of phenomena that share a common feature (Kuehn 2011, page 1029): “The noise induces a behavior in a system that cannot be found in the deterministic version.”

We investigate the use of nonparametric estimated 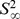 and the diffusion function g(.) as early warning indicators (Table 1). One could also consider the drift function 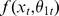 of equation [1] as an indicator. The derivative df(.)/dx evaluated at a deterministic steady state is an approximation of the eigenvalue of the linearization around that deterministic steady state, which becomes zero at the critical transition point. However, we focus here on the indicators based on variance, 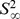 and g(.). If we observe time series, 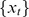, we can estimate 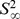, f(.) and g(.) using the estimators explained below. It is also possible to compute nonparametric estimates of higher moments (Carpenter and Brock 2011) [22] but in the interest of brevity we do not address higher moments here.

**Table 1:** Indicators considered in this paper.

Name of indicator	Interpretation	Symbol	NonparametricEstimate
Conditionalvariance	Variance of x as a function of x. We use the conditional variance as an approximation of thelong-run stationary variance of x, 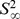 . In a critical transition caused by a local co-dimensionone bifurcation of the drift function, the long-run stationary variance of the linearizationaround the deterministic steady state becomes infinite at the transition point. The long runstationary variance may increase as a noise-induced transition is approached.	S^2^	Equation [5]
Diffusion	Variance of dx as a function of x. We use the estimator as an approximation of 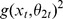 as defined near equation [1]. In a noise-induced transition, the variance matrix functiong’g (or g^2^) increases as the transition is approached.	g’g, or g^2^	Equation [7]

### Nonparametric Estimators

We estimate the indicators using nonparametric regression [23], [24]. There are many different approaches to analysis of data sets generated by stochastic dynamical systems like [1] besides the nonparametric regression approach taken here (for example Siegert et al. [25] for approaches based upon the Fokker Planck equation of [1]). We use the nonparametric approach of Bandi and Phillips [26] and their references here because it can approximate the shape of [1] without the need to specify a functional form.

The quantities to be estimated as functions of x (or y depending on the application) are the conditional variance 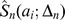 (defined in [5] below), drift f(.), and diffusion g(.). We use symbols like dx or dW to denote infinitesimals and we use dx1 or dxd to denote d dimensional column vectors and d by d dimensional square matrices respectively. For data analysis we represent the dynamics using equations [1] and [2] where x and f are dx1 vectors, g is a dxd square matrix, 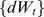, is an dx1 vector of Wiener processes, 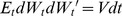, and 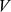 denotes a dxd positive definite square matrix. Data are sampled over the time interval [0,T], T finite, at equi-spaced times 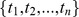. Thus we have n observations on the process 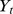, denoted by 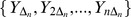 at 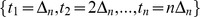,where 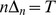.

Consider a vector *a* of equi-spaced values of *Y* for which the estimators will be computed. Let 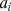 denote the i’th component of this vector. The conditional variance function, 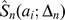, is estimated as the difference between the second conditional moment and the square of the first conditional moment as follows,
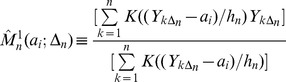
(3)

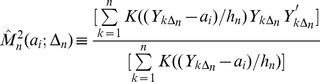
(4)

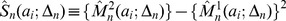
(5)


Here 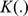 is a kernel function of bandwidth 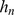 defined in [8] below (for the multivariate case) or [9] below (for the univariate case). Equation [3] is the kernel-weighted average of the first power of Y. Equation [4] is the kernel-weighted average of the second power of Y. Expressions [3] and [4] are standard nonparametric regression conditional moments estimators of the first conditional moment and the second conditional moment for the one dimensional case [24]. The dx1 vector of first moments and the dxd matrix of second moments for the d-dimensional case are computed by an obvious expansion of [3]–[5] [24].

Estimator [5] is a standard nonparametric estimator of the conditional variance. From this point on we use the simple notation 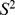 in place of 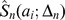. Equations [3]–[5] are known to be consistent estimators under regularity conditions [23], [24] (Chapter 7) that include strict stationarity and ergodicity, which we assume are satisfied by [1].

Nonparametric estimates of drift and diffusion can be computed as a function of any variable that is measured at the same times as *Y,* subject to regularity conditions specified in Bandi and Phillips (2010). In the case Y = X, for a particular element *a_i_* the estimator for the dx1 vector of *f(.)* is given by
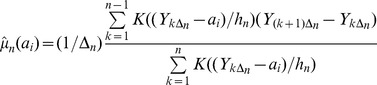
(6)where 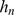 is a bandwidth [26]. Equation [6] is the kernel-weighted average of the first difference of Y. Thus 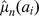 estimates the vector 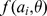. When Y = c’X, [6] estimates the vector 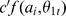.

The estimator for the dxd matrix of second moments, corresponding to the covariance matrix for *g(.)* is given by
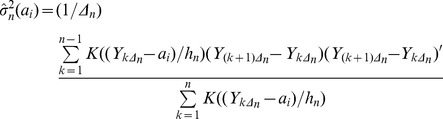
(7)where the apostrophe denotes transpose. Equation [7] is the kernel-weighted average of the square of the first difference of Y. If one sets Y = X in equation [7] then the matrix 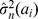 estimates the matrix 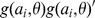. When Y = c’X, [7] estimates the matrix, 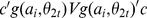. Bandi and Phillips [26] review literature that locates sufficient conditions for [7] to be a strongly consistent estimator of the matrix 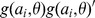, i.e. for [7] to converge with probability one to 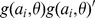.

Following Bandi and Phillips [26] (equations (48) and (49)) for any bandwidth 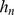, and any dx1 vector z, 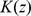 is defined by
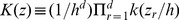
(8)where k(.) is any one dimensional kernel function that satisfies their regularity conditions. The product kernel [8] where z is the dx1 vector 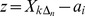 is useful for multivariate applications. When Y = c’X where the dimension d’ of Y is less than or equal to d, then the dimension of z will be d’.The Gaussian kernel function we used for one dimensional estimation is



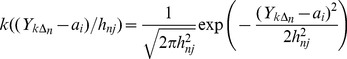
(9)For multivariate kernels insert [9] into [8] after replacing the vectors by their j’th components, and run j = 1,2,…,d and j = 1,2,…,d’ for Y = X and Y = c’X respectively.

Drift and diffusion estimates are smoother the larger the bandwidth. Johannes [27] provides advice on choice of bandwidth. Methods for computing an optimal bandwidth exist [23]. We have found that these methods undersmooth the drift and diffusion functions, yielding curves that are too irregular in experiments where the drift and diffusion functions are known a priori. Therefore we prefer the guidelines of Johannes [27] which scale the bandwidth to the standard deviation of the time series.

Bandi and Phillips [26] review literature that shows that as one samples more and more frequently within a fixed interval [0,T] the estimate of the matrix 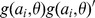 becomes infinitely precise whereas the precision of the drift remains low. But in order to drive the variance of the drift to zero one must increase the length of the sampling interval [0,T] to infinity. We call sampling by sending T to infinity “long span sampling” and sampling more often within the interval [0,T] “infill sampling”. Infill sampling within each interval of time achieves a much more precise estimate of the moment matrix function gg’ than the drift function f whereas long span sampling is needed to get a precise estimate of the drift function f. Simulations that illustrate this point are available in the literature [22], [27], [28].

### Simulation Studies

We used two well-studied ecosystem models as case studies. The lake eutrophication model [6] in one dimension is
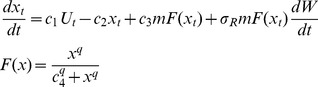
(10)


The regime shift is driven by a slow increase in the mass of phosphorus in the watershed soil U_i_. Phosphorus in sediment m is constant at 200 g m^−2^. Other fixed parameters are input coefficient c_1_ = 0.00115, output coefficient c_2_ = 0.85, recycling rate coefficient c_3_ = 0.019, recycling half-saturation coefficient c_4_ = 2.4, exponent in the recycling function q = 8, and standard deviation of Wiener shocks (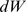) to recycling σ_R_ = 0.005. For simulations reported here, Δ = 0.1 and N = 10,000. The soil phosphorus U was increased linearly from 600 to 1100 g m^−2^ over the N time steps.

In equation [10], noise is added to the recycling parameter, such that the recycling term is 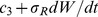. As a result the diffusion 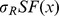
is a function of x. As an instructive contrast we also considered the additive noise case
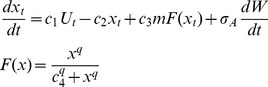
(11)


In [11], recycling is deterministic, and diffusion is constant 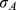.

To evaluate the performance of low-dimensional indicators for a higher-dimensional system, we analyzed a 5-dimensional model of a lake food web [29] using samples of one or three components. This case study illustrates the performance of the estimators when only part of a multi-dimensional system is sampled. Details of the model are presented in Supporting Information S2. The model describes the dynamics of adult and juvenile piscivorous fishes, planktivorous fishes, herbivorous zooplankton and phytoplankton as driven by nutrient inputs, irradiance, and harvest of adult piscivores. As the harvest rate of adult piscivores is increased slowly, the system undergoes a bifurcation that is announced in advance by rising variance [29].

We performed two kinds of experiments with the 5-dimensional model. In both cases we gradually increased the bifurcation parameter (harvest rate of adult piscivores). In the first case, we assumed that data were available only for phytoplankton, a one-dimensional data set. In the second case, we assumed that data were available for phytoplankton, zooplankton, and planktivorous fishes.

A R function for computing the indicators is presented in Supporting Information S3.

For each model, three types of simulations are presented. (1) To show estimates over a wide range of the observed state variable, simulations that extend before and after the critical transition were analyzed by computing the estimators. (2) Early warnings, to be useful, must be detected prior to the critical transition. To investigate early detection, we terminated simulations prior to the critical transition and computed the estimators. (3) In order to assess the precision of the estimators, we conducted Monte Carlo simulations. For each Monte Carlo replicate, time series were computed up to the critical transition, but not beyond, and the estimators were then calculated. Then the functions for conditional variance, drift and diffusion were averaged over 1000 realizations to compute the mean and standard deviation. For univariate analyses, nonparametric functions were computed on a mesh of 500 values spanning the range of the observed state variable using a bandwidth of 0.3 times the standard deviation of the entire series. In addition to the three types of univariate simulations, we present a multivariate example for the food web model. The multivariate example was computed in three dimensions, planktivore, herbivore, and phytoplankton, on a mesh of 75×75×75 values using a bandwidth of 0.4 times the standard deviation of the entire series.

In order to be useful, an early warning indicator must be plotted against time. The nonparametric methods yield indicators as a function of the observed state variable. We used linear interpolation to obtain indicator values as a function of time, by interpolating indicator values for each observed value of the state variable. Because the mesh used for the nonparametric computations is quite dense, linear interpolation is likely to be reasonably accurate.

## Results

**Figure 1:**
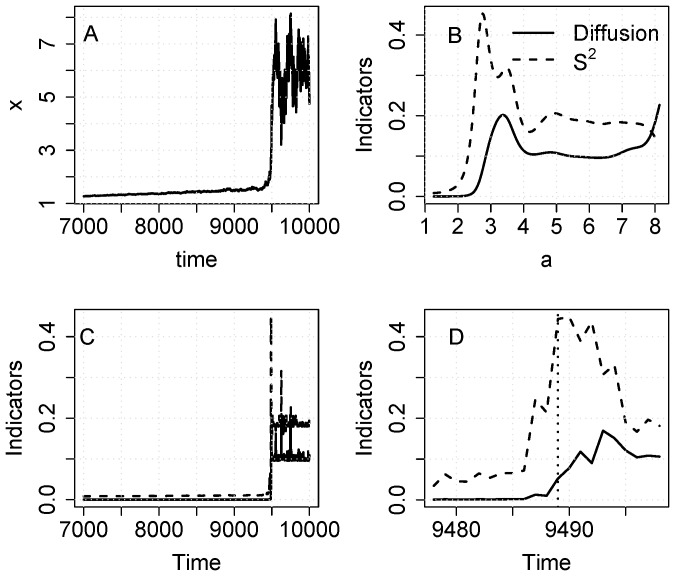
Simulation with the eutrophication model using noise added to recycling (eq. 10). A. Time series of the state variable. Note that the sample includes data before and after the transition. B. Diffusion and S^2^ versus a. C. Diffusion and S^2^ versus time. D. Diffusion and S^2^ versus time for a short time interval near the transition.

As an initial demonstration of the indicators, we analyze the eutrophication model with noise added to recycling (equation [10]). The time series shows clearly that x (lake phosphorus in this case) increases in level and fluctuates more after the transition point (Fig. 1A). Plots of the indicators versus a show a sharp increase in S^2^ as a rises near 3 (Fig. 1B) Diffusion also rises as a approaches 3, and then tends to plateau. Plots of the indicators versus time show that the increases are quite sharp near the inflection point of the time series (Fig. 1C). When the graph zooms in on events near the inflection point, it is clear that S^2^ increases at least 5 time steps prior to the inflection point whereas diffusion increases much closer to the inflection point (Fig. 1D).

**Figure 2:**
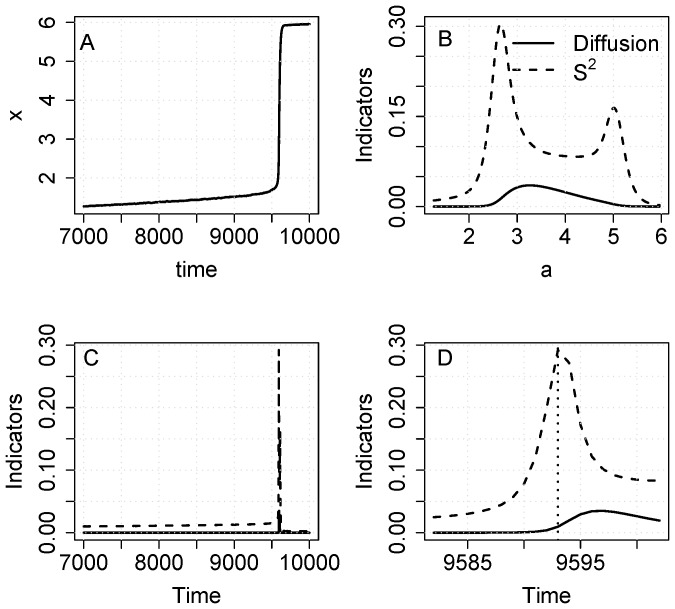
Simulation with the eutrophication model using additive noise (eq. 11). A. time series of the state variable. Note that the sample includes data before and after the transition. B. Diffusion and S^2^ versus a. C. Diffusion and S^2^ versus time. D. Diffusion and S^2^ versus time for a short time interval near the transition.

When noise is purely additive (equation [11]), diffusion should be constant when plotted versus a or time. An additive-noise example is presented in Fig. 2. The change in level is clear but any changes in variability are subtle (Fig. 2A). As expected, S^2^ rises prior to the inflection point near a = 3 (Fig. 2B). After the inflection point there is a small increase in diffusion that gradually declines at values of a ≥ 3.5. Both S^2^ and diffusion spike near the inflection point when plotted versus time (Fig. 2C). Zooming in on the inflection point, the increase in S^2^ starts several time steps prior to the inflection point whereas the increase in diffusion occurs after the inflection point.

The increase in diffusion seen in Fig. 2 appears to be an artifact of averaging during the steep rise in the time series. The elevated values of diffusion occur for x values roughly between 2.5 and 5, which occur for only a few data points during the steep rise of the time series. Apparently the bandwidth is of a size that leads to a small increase in the diffusion estimator during the steep rise. When the time series settles down around the higher equilibrium near x ≈ 6, the diffusion estimate is again near zero.

**Figure 3:**
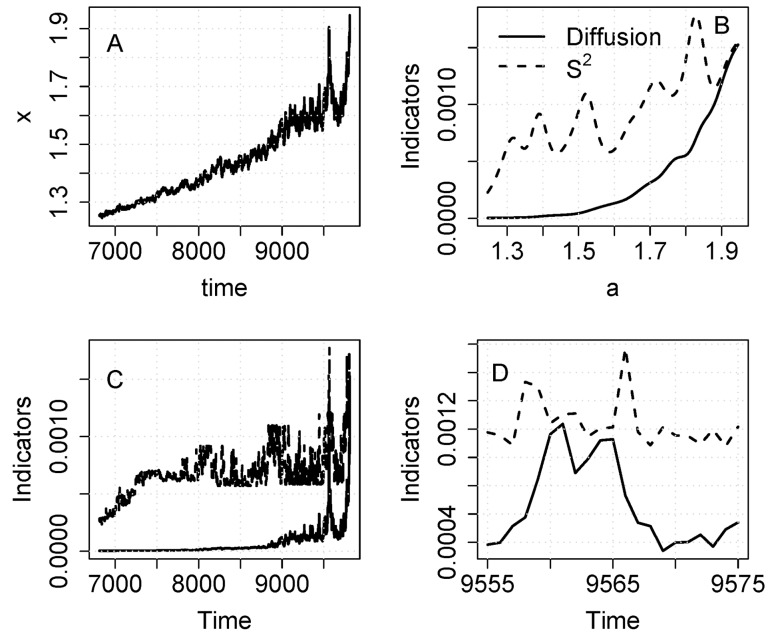
Simulation with the eutrophication model using noise added to recycling (eq. 10). A. Time series of the state variable. Note that the sample includes data up to, but not after, the transition. B. Diffusion and S^2^ versus a. C. Diffusion and S^2^ versus time. D. Diffusion and S^2^ versus time for a short time interval near the transition.

To be useful as early warning signals, the indicators must increase as the ecosystem approaches the transition, using data measured only up to some point in time prior to the transition. Fig. 3 presents an example (Fig. 3A). In this realization, both S^2^ and diffusion show increases with a (Fig. 3B). Also, increases in both indicators are apparent in plots versus time (Figs. 3C, D).

**Figure 4:**
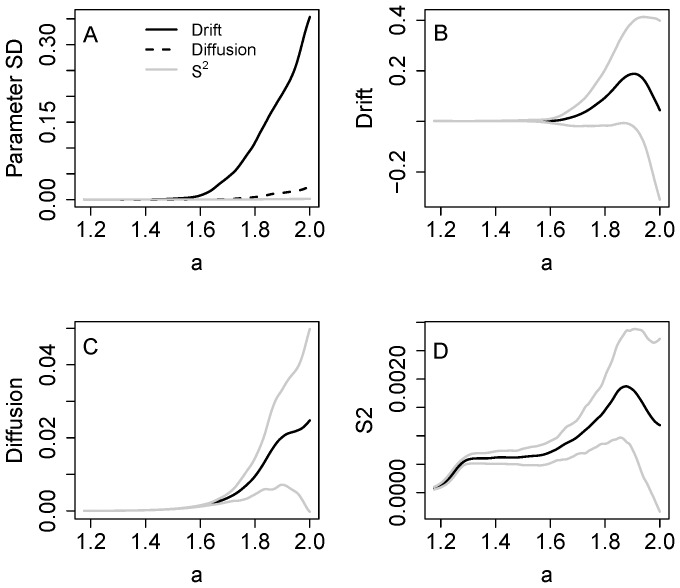
Monte Carlo simulations with the eutrophication model using noise added to recycling (eq. 10). The samples ended at x = 2. A. Parameter standard deviations for drift, diffusion and S^2^ versus x. B. Drift ± standard deviation versus a. C. Diffusion ± standard deviation versus a. D. S^2^ ± standard deviation versus a.

Uncertainties become relatively large if the data do not span the transition. Fig. 4 presents Monte Carlo simulations with noise added to recycling (equation [10]) for 1000 time series, each of which was truncated at x = 2. The standard deviation of drift is very large, while those of diffusion and S^2^ are smaller (Fig. 4A). Confidence bands for drift span zero, indicating that even the sign is uncertain, for values of a above about 1.6 (Figs. 4B). Both diffusion and S^2^ are clearly positive up to a values of about 1.9 or even larger (Figs. 4C, D).

**Figure 5:**
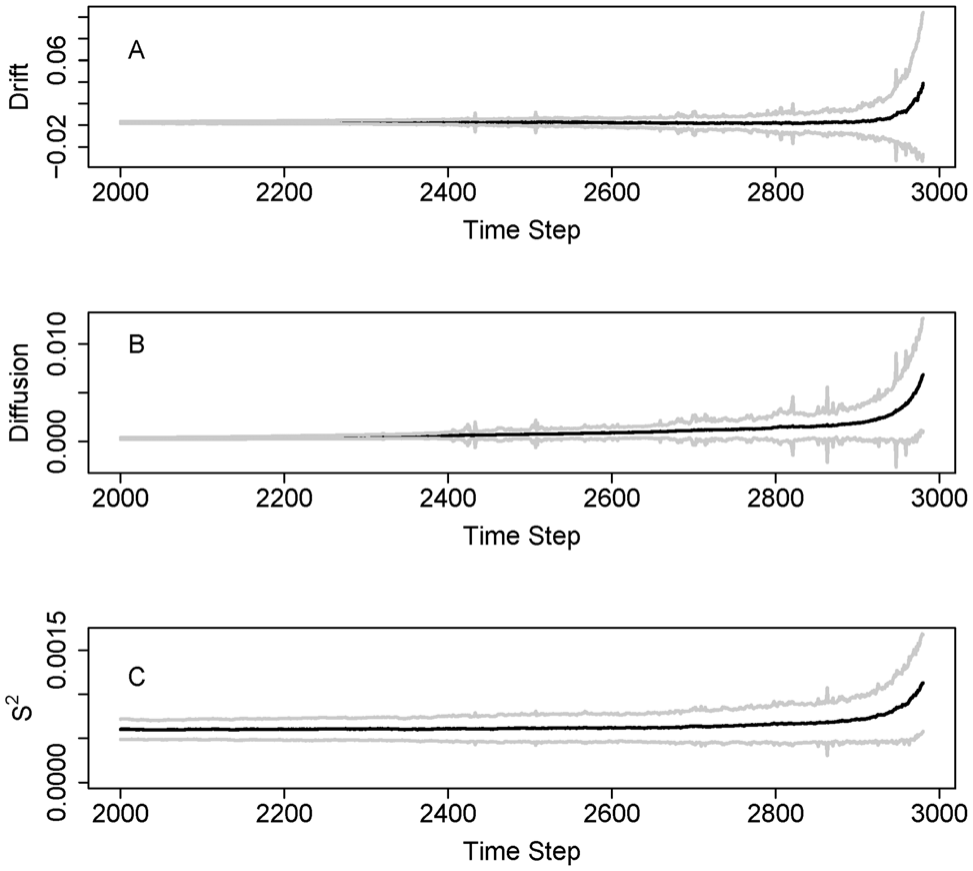
Time series plots with confidence bands (± standard deviation) for the eutrophication model, interpolated from the functions in Fig. 4. A. Drift. B. Diffusion. C. Conditional variance S^2^.

Plots of indicators and confidence bands versus time show that uncertainties increase as the indicators approach the transition point (Fig. 5). Time plots give a somewhat different impression from plots versus a, because not all a values occur in the time series. This is especially true for drift which is not discernibly different from zero over many time steps (Fig. 5A). However the confidence band for diffusion also includes zero for some time steps (Fig. 5B). Conditional variance S^2^ is generally larger than zero over time (Fig 5C).

**Figure 6:**
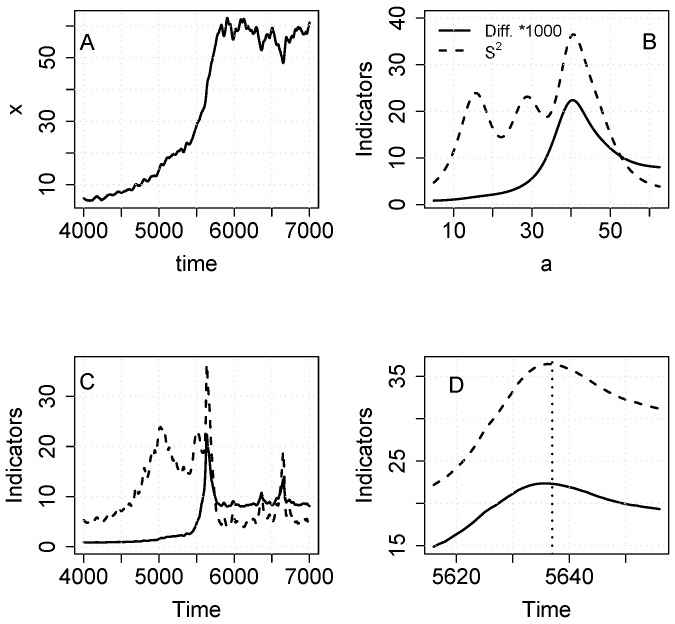
Simulation with the food web model (Supporting Information S2). A. Time series of phytoplankton. Note that the sample includes data before and after the transition. B. Diffusion (times 1000) and S^2^ versus a. C. Diffusion (times 1000) and S^2^ versus time. D. Diffusion (times 1000) and S^2^ versus time for a short time interval near the transition.

From a single realization of the lake food web model (Supporting Information S2), we analyze the time series for phytoplankton (Fig. 6A). The inflection point occurs around time step 5624 which corresponds to X ≈ 40. Both S^2^ and diffusion increase at values of a prior to the critical transition (Fig. 5B). When the indicators are plotted against time, there is a notable increase in S^2^ and smaller increase in diffusion prior to the inflection point (Fig. 6C, D).

**Figure 7:**
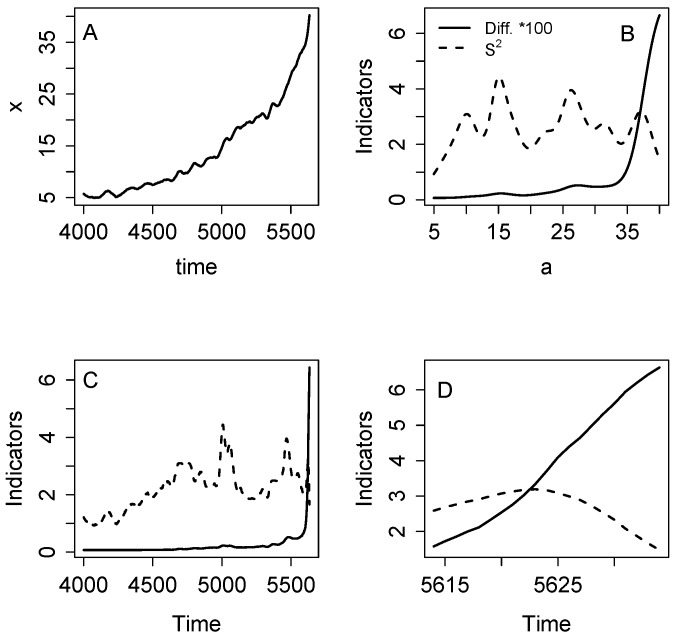
Simulation with the food web model (Supporting Information S2). A. Time series of phytoplankton sampled up to x = 40, just before the transition. B. Diffusion (times 100) and S^2^ versus a. C. Diffusion (times 100) and S^2^ versus time. D. Diffusion (times 100) and S^2^ versus time for a short time interval near the transition.

To illustrate the use of the indicators as an early warning before the critical transition in the food web model, we present results for a time series that ended when x = 40 (Fig. 7A). S^2^ is high and fluctuating for a ≥ 10, and diffusion increases for a ≥ 35 (Fig. 7B). The plot of indicators versus time shows that S^2^ is elevated over much of the range, whereas diffusion increases only as the system gets close to the transition point (Fig. 7C). Zooming into the last 20 time steps, it is evident that diffusion continues to rise whereas S^2^ actually declines in the last few time steps (Fig. 6D). As noted in Supporting Information S1, the relationship of S^2^ and diffusion is not necessarily monotonic.

**Figure 8:**
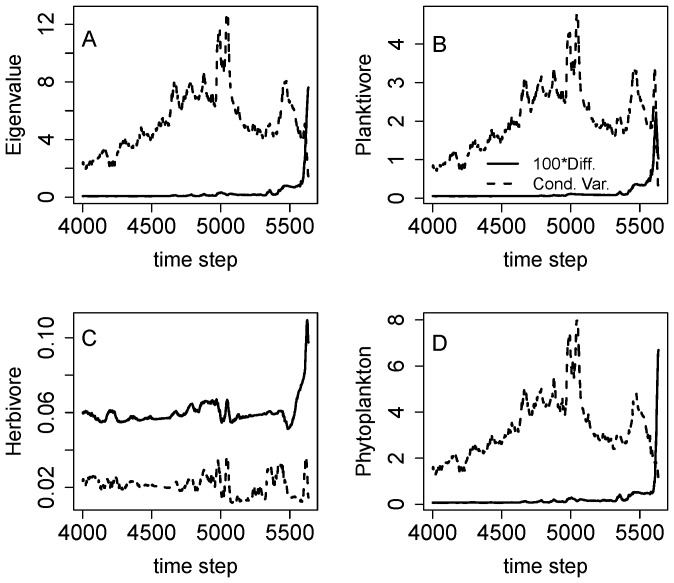
Results of multivariate nonparametric analysis of the same food web simulation depicted in Fig. 7. For multivariate analysis, time series for planktivore, herbivore and phytoplankton were analyzed. Time series are presented for 100*diffusion (solid line) and conditional variance (dashed line). (A) Largest eigenvalue of the diffusion and conditional variance matrices. (B) Variance of planktivores from the diffusion and conditional variance matrices. (C) Variance of herbivores from the diffusion and conditional variance matrices. (D) Variance of phytoplankton from the diffusion and conditional variance matrices.

To evaluate the information that might be gained by multivariate analysis, we computed multivariate indicators using the planktivore, herbivore and phytoplankton time series simultaneously. For each indicator, multivariate analysis yields a 3×3×3 array of estimated values. This array is very difficult to visualize and understand. However, over time the ecosystem took a one-dimensional path through this high-dimensional space. Therefore, time plots of the indicators were constructed by interpolation (Fig. 8). The multivariate analysis was also slow to compute. For 1635 data points in three dimensions, computing the indicators on the 75×75×75 mesh took 21.4 hours on a Intel 1.6 GHz processor running Windows XP and R 2.10.1, downloaded 2009-12-14.

The maximum eigenvalue of the conditional covariance matrix and the conditional covariances of the planktivore and phytoplankton show similar patterns with peaks near time step 5000 (Figs. 8 A, B, D). The conditional variance for the herbivore (Fig. 8C) behaves differently, with some fluctuations near time step 5000 but no distinct peak. In the model, herbivores are stabilized by a refuge and this factor may account for the muted responses of herbivores [29]. All indicators for diffusion show sharp rises near the end of the time series (Figs 8A–D). The difference in responses suggest that the regime shift for the system as a whole, indicated by conditional variance, occurred around time step 5000 whereas a critical transition in g() occurs near the end of the sampled series.

The responses of the conditional variance and diffusion for phytoplankton are roughly similar for the univariate and multivariate analyses (compare Fig. 7C with Fig. 8D). We would not expect them to be identical, because they employ different bandwidths and different meshes for a, due to the slow computation speed of the multivariate method. The phytoplankton responses are quite similar to the eigenvalues which reflect the integrated response of the entire ecosystem. Thus in this case a univariate analysis of phytoplankton would have been sufficient as an early warning.

**Figure 9:**
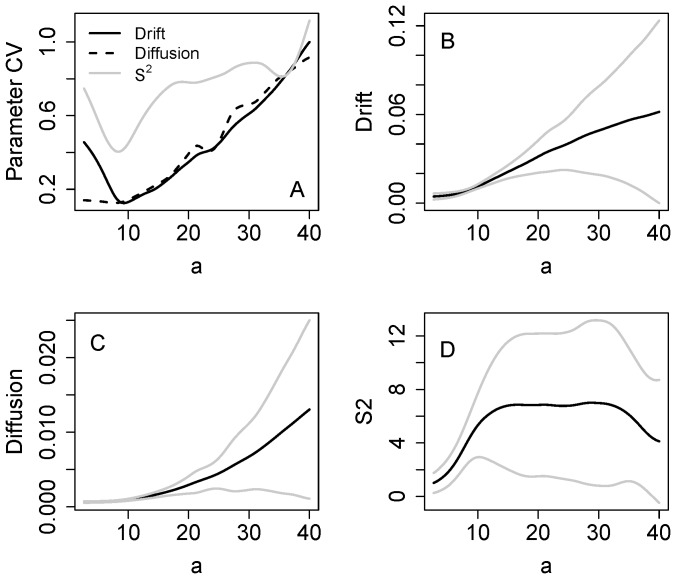
Monte Carlo simulations with the Food Web model (Supporting Information S2) showing results for phytoplankton. The samples included data for X ≤ 40, just below the transition. A. Parameter standard deviations for drift, diffusion and S^2^ versus a. B. Drift ± standard deviation versus a. C. Diffusion ± standard deviation versus a. D. S^2^ ± standard deviation versus a.

Precision of the estimators computed from 1000 Monte Carlo samples of the food web model showed high uncertainty as the state variable approaches the transition point (Fig. 9A). When the observed state variable is near 40, Confidence bands overlap zero for all of the indicators (Fig. 9B–D).

**Figure 10:**
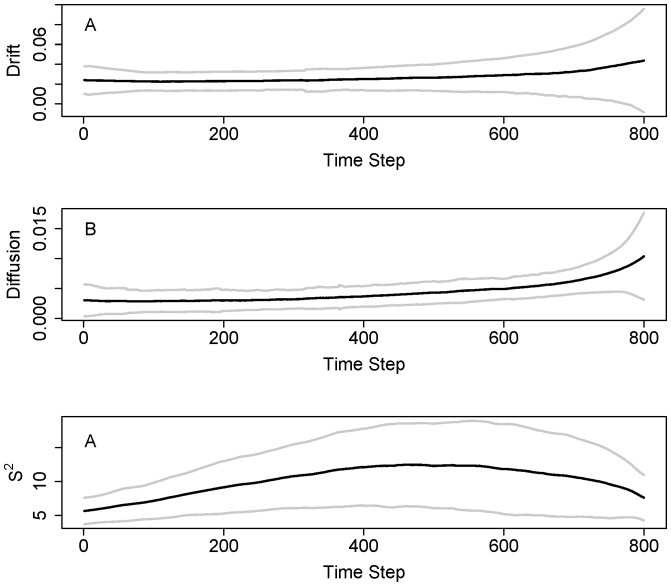
Time series plots with confidence bands (± standard deviation) for the food web model, interpolated from the functions in Fig. 9. A. Drift. B. Diffusion. C. Conditional variance S^2^.

Time plots of the Monte Carlo sample show that confidence bands are highest near the transitions (Fig. 10). Drift is often close to zero (Fig. 10A). Diffusion is non-zero and generally rises across the time window (Fig. 10B). Conditional variance is consistently different from zero, and is highest near the middle of the time frame (Fig. 10C). This pattern is similar to what we observed in the analyses of single time series. The time axis is re-scaled in Fig. 10 in order to put all of the time series on the same basis relative to the inflection point.

## Discussion

Estimators of S^2^ and diffusion are clearly elevated near the critical transition in time series that are sampled both before and after the transition (Figs. 1, 5). Such time series are a case of long-span sampling. According to theory and previous simulation studies, long-span sampling is needed to obtain precise estimates of drift [30].

Comparison of the noise-in-parameter (Fig. 1) and additive noise (Fig. 2) cases for the eutrophication model shows clear differences related to the fact that diffusion is constant in the additive case. Nonetheless S^2^ is strongly elevated around the critical transition even in the additive case. The rise in S^2^ suggests a bifurcation in either f() or g(), whereas a rise in the diffusion function suggests a bifurcation in g(). The strong response of S^2^, combined with the weak and transient response of diffusion, could be used to discriminate noise-induced transitions (such as that generated by eq. [10]) from drift-induced transitions (such as that generated by eq. [11]). For example, the current discussion about the role of noise-induced transitions in the Dansgaard-Oeschger events of climate change history [31], [32] could perhaps be addressed using nonparametric methods to measure the conditional variance and diffusion functions.

To serve as early warnings, the indicators must yield signals before the regime shift occurs (Figs. 3, 6). In early warning settings, long-span sampling may be impossible because only data prior to the transition are useful for early warnings. Nonetheless, infill sampling can be used and this will improve the precision of diffusion estimates.

Single realizations of the eutrophication and food web models show early warnings in the estimates of S^2^ and diffusion for data collected before the transition (Figs. 3,6). These increases, while notable, are less pronounced that those we observed using rolling window statistics for the same models [6], [29]. However it is difficult to compute unambiguous error estimates for rolling window analyses. For the nonparametric method, Monte Carlo analysis shows that estimates are surrounded by considerable uncertainty (Figs. 4, 7). For drift, even the sign is unknown. For the eutrophication model (Fig. 4), there is a maximum near a = 1.9 which could indicate an eigenvalue of zero (the eigenvalue is the derivative of drift with respect to a). Because of the wide confidence band, however, any estimate of the eigenvalue is highly uncertain. Both S^2^ and diffusion are clearly positive and increasing prior to the transition. However, the confidence bands of S^2^ and diffusion span zero at the highest values of a. Apparently it is difficult to obtain precise estimates of these functions at extreme values of the data. Nonetheless, for the great majority of stochastic realizations the estimates of S^2^ and diffusion increase before the transition and thereby provide useful early warnings.

Many previous studies of early warning indicators have employed time series of autocorrelation, variance, and other statistics computed on a sequence of rolling-window subsets of the data [8], [29]. Our analyses have several interesting implications for rolling-window statistics. (1) The autocorrelation and the variance are both affected by both drift and diffusion (Supporting Information S1). Therefore it is not possible to separate drift and diffusion components using these statistics. However, nonparametric analysis does provide a means of estimating drift and diffusion separately. This is advantageous where researchers seek to distinguish additive noise from nonlinear noise. (2) The nonparametric estimate of S^2^ is analogous to rolling-window variance. Our analyses corroborate the value of variance as an early-warning indicator. (3) Monte Carlo analyses indicate that trajectories of S^2^ and (for noise-induced transitions) diffusion will generally increase as a critical transition is approached. However, uncertainties of both indicators tend to become large as the system approaches the transition. In some cases it may be possible to increase the precision of diffusion estimates by sampling more frequently. Nonetheless, while these indicators generally rise prior to a critical transition this pattern is not guaranteed. Decreases or cycles are possible when the transition is near.

There are important ecological cases where an appropriate structural model (specific form of [1]) is known from other research or can be determined by fitting models to the data. For example, extensive research on many ecosystems for many years suggests certain structural forms for well known regime shifts in lakes [2], [33]. In other cases methods for optimal model identification can be used to infer the structural form of nonlinear models for ecological time series [15], [16]. These approaches have the powerful advantage of strong inference about the underlying nature of the bifurcation. We are enthusiastic about early warnings based on structural model identification, but we recognize that in many cases the data will not be up to the task. Structural models may not always be available in ecological applications because long time series, typically including covariates and several instances of regime shift, are needed to fit nonlinear models of bifurcations [2]. Furthermore, the time to relax to the stationary distribution can be very long especially as the bifurcation parameter gets close to the critical value of the bifurcation parameter [20], [21], [34]. Thus even if the bifurcation parameter 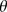 is known and can be estimated, detection of tiny changes as 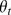 gets close to the critical value 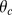 may not be the most efficient approach to constructing early warning indicators. Therefore we focus here on alternatives that are useful even when the structural model cannot be identified.

The nonparametric method will typically require more observations than a parametric method. This is so because parametric methods can exploit the knowledge embedded in parametric specification of components of equation [1]. However the parametric assumptions may be invalid and thereby invalidate the results of the parametric method. Hence there is a tradeoff between reducing the risk from mistaken model specification in parametric methods and the heavy data demands of the nonparametric method. Different situations may be best suited to one approach or the other. If data are plentiful and measured at high frequencies, then the nonparametric method provides a rather precise estimate of the conditional variance and diffusion terms [35]. High frequency data are increasingly common in ecology. In addition. one can learn about noise-induced versus non-noise-induced transitions by comparing S^2^ and diffusion functions.

In order to be useful, early warning indicators must be plotted against time. Strictly speaking, nonparametric functions should be fitted against random covariates such as the observed state variable, and not time. However, interpolation can be used to provide a time series of the indicators as we have shown here.

Nonparametrics are expensive to compute even for a coarse mesh in the multivariate case. However, our results show that even one dimension sampled from a multi-dimensional system can provide an early warning.

Error estimates for nonparametric estimates are a topic for further research. For cases where the underlying structural model is not known, Monte Carlo analysis using the fitted functions is difficult because drift is highly uncertain. While Monte Carlo analysis using the fitted function is beyond the scope of this paper, we suggest that it may be informative to guess some plausible structural models and compute Monte Carlo analyses to get a rough idea of how large the uncertainties could be.

In management applications, one would compute the nonparametric functions at regular intervals of time to check for increases in conditional variance or diffusion. Monte Carlo analyses presented here show that estimates of the functions become rather uncertain near the critical transition point. While the majority of the Monte Carlo realizations suggest an impending transition, some do not. There will, therefore, be cases where the early warning is not detected even though a critical transition is imminent. In management decisions, an imperfect early warning is more valuable than no warning at all. Even an uncertain early warning may have great value when there is risk of a very expensive regime shift.

There are important ecological cases where an appropriate structural model (specific form of [1]) is known from other research or can be determined by fitting models to the data. For example, extensive research on many ecosystems for many years suggests certain structural forms for well known regime shifts in lakes [2], [33]. In other cases methods for optimal model identification can be used to infer the structural form of nonlinear models for ecological time series [15], [16]. These approaches have the powerful advantage of strong inference about the underlying nature of the bifurcation. We are enthusiastic about early warnings based on structural model identification, but we recognize that in many cases the data will not be up to the task. Structural models may not always be available in ecological applications because long time series, typically including covariates and several instances of regime shift, are needed to fit nonlinear models of bifurcations [2]. Furthermore, the time to relax to the stationary distribution can be very long especially as the bifurcation parameter gets close to the critical value of the bifurcation parameter [20], [21], [34]. Thus even if the bifurcation parameter 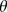 is known and can be estimated, detection of tiny changes as 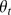 gets close to the critical value 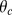 may not be the most efficient approach to constructing early warning indicators. Therefore we focus here on alternatives that are useful even when the structural model cannot be identified.

The need for early warning indicators of ecological regime shifts, despite uncertainty about the true data generating process, raises many challenges. Nonetheless, this paper provides several reasons to think that early warning indicators for ecosystem regime shifts are worth pursuing, even if there is little information about underlying processes. Nonparametric methods that assume no particular data generating process (other than that it is a stochastic differential equation like [1]) can yield useful estimates of the conditional variance and diffusion functions. In principle, such estimates could be updated periodically over time to track the movement of the system toward or away from a critical transition. Nonparametric methods are especially effective for high-frequency automated observations that are becoming more available in ecology.

The encouraging exploratory results presented here suggest that further research to understand and improve ecological early warning indicators is worth the effort.

## Supporting Information

Figure S1 **State variables versus time for a realization of the food web model illustrating dynamics of the five dimensions.** Adult and juvenile piscivore curves were multiplied by 4 for convenient display on the same axes as the other variables.
(TIF)

Supporting Information S1 (DOCX)

Supporting Information S2 (DOCX)

Supporting Information S3 (DOCX)
