# Supplementary figures and images for: Early Warnings of Regime Shift When the Ecosystem Structure Is Unknown
Source: PLoS One. 2012 Sep 21;7(9):e45586. doi: 10.1371/journal.pone.0045586 (PMC3448650; doi:10.1371/journal.pone.0045586)

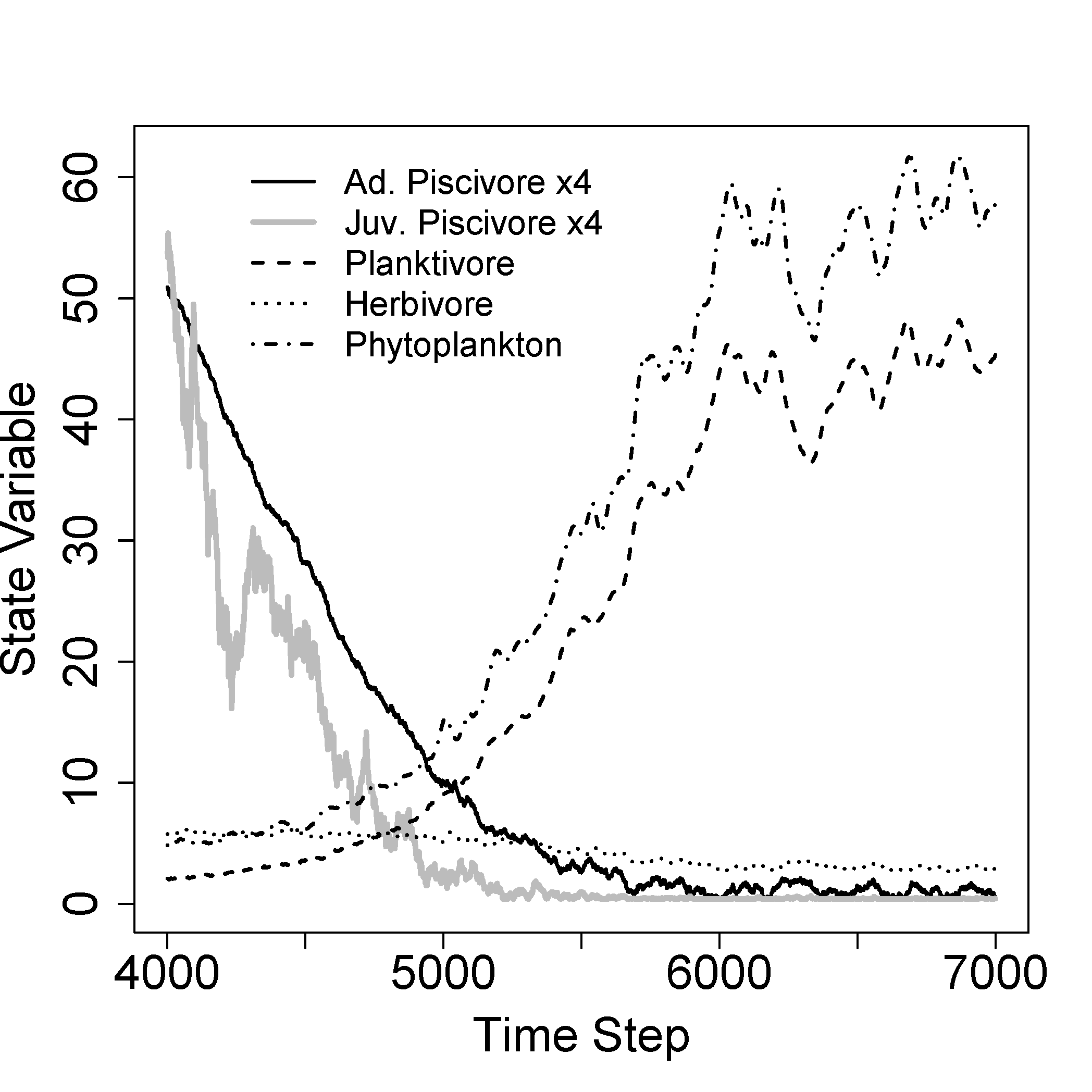

Supplement: Figure S1 — State variables versus time for a realization of the food web model illustrating dynamics of the five dimensions. Adult and juvenile piscivore curves were multiplied by 4 for convenient display on the same axes as the other variables. (TIF) [file pone.0045586.s001.tif]
