# Supplementary material for: Early Warnings of Regime Shift When the Ecosystem Structure Is Unknown
Source: PLoS One. 2012 Sep 21;7(9):e45586. doi: 10.1371/journal.pone.0045586 (PMC3448650; doi:10.1371/journal.pone.0045586)
Supplement: Supporting Information S1 — (DOCX) [file pone.0045586.s002.docx]

*Supporting Information 1. Background*

This Appendix summarizes theoretical results about measures of autocorrelation and variance as equation [1] approaches a critical transition. For readers who wish to skip the technical background, here are the key points. For many critical transitions of ecological interest, autocorrelation is one and variance is infinity at the critical point. The pathway to the critical point may or may not be monotonic, for both autocorrelation and variance. Trajectories of both autocorrelation and variance depend on the process *f(.)* and noise *g(.)* of equation [1] in complicated ways that may differ from ecosystem to ecosystem. Below we develop these points in more detail.

Assume $\theta$ does not change over time. To simplify notation we drop subscripts on $\theta's$ in the drift and diffusion functions. To evaluate stability, we linearize [1] around a deterministic steady state by putting $g(x,\theta)=\varepsilon g_{0}(x,\theta),\varepsilon>0$ in [1] doing a small noise expansion [[1](#_ENREF_1)]. Let $\bar{x}(\theta)$ be a deterministic steady state for [1]. I.e. $f(\bar{x}(\theta),\theta)=0$. Expand [1] in a Taylor series around $x=\bar{x}(\theta),\varepsilon=0$, and drop higher order terms about this deterministic steady state. Let $\delta x_{t}\equiv x_{t}-\bar{x}(\theta)$. In more detail note that the term

$$\varepsilon g_{0}(\bar{x}(\theta)+\delta x_{t},\theta)dW_{t}=(\varepsilon g_{0}(\bar{x}(\theta),\theta)+\varepsilon g_{0x}(\bar{x}(\theta),\theta)\delta x_{t})dW_{t}=\varepsilon g_{0}(\bar{x}(\theta),\theta)dW_{t}+H.O.T.$$

because $\varepsilon\delta x_{t}dW_{t}$ is higher order in $(\delta x_{t},\varepsilon)$. Here “H.O.T.” denotes “Higher Order Terms.”

We obtain the following first order approximation in $\delta x_{t}\equiv x_{t}-\bar{x}(\theta)$,

$d(\delta x_{t})=f_{x}(\bar{x}(\theta),\theta)\delta x_{t}dt+g(\bar{x}(\theta),\theta)dW_{t}$ [A.1.1]

Note that $f_{x}(\bar{x}(\theta),\theta),\text{ }g(\bar{x}(\theta),\theta)$ are dxd matrices, where $f_{x}(\bar{x}(\theta),\theta)$ denotes the dxd derivative matrix. A bifurcation in the drift function f occurs as $\theta\to\theta_{c}$ when the real part (real parts of the leading pair in the complex case) of the leading eigenvalue(s) of $f_{x}(\bar{x}(\theta),\theta)$ become positive.

Biggs et al. [[2](#_ENREF_2)] (Appendix) show that at a critical point for [A.1.1] the steady-state autocorrelation is one and the steady-state variance is infinity even when one only observes the one dimensional indicator, $\delta y_{t}=c'\delta x_{t}$. This follows from the fluctuation dissipation theorem [[3](#_ENREF_3)]. The exposition in the Appendix to Biggs et al. [[2](#_ENREF_2)]is especially tailored to the applications discussed here. Both continuous-time and discrete-time cases are analyzed by Biggs et al. [[2](#_ENREF_2)]. To show how the autocorrelation and variance are related to [A.1.1], we present the discrete time case. We focus here on discrete time because we also want to investigate how shortening the length of the time over which first order autocorrelation is estimated impacts the signal. Our main point – that both autocorrelation and variance depend on both *f(.)* and *g(.)* – also holds in continuous time.

The discrete-time analog of [A.1.1] is

$x_{t+1}=Ax_{t}+Ge_{t+1}$ [A.1.2]

where x is a vector of deviations from steady-state, {e_t_} is a sequence of IID noise with mean zero and unit covariance matrix, the process matrix A is a square nxn matrix and the noise matrix G is an nxm matrix.. The long-run covariance matrix for x is (for both discrete time and continuous time cases for ease of comparison)

$S_{\infty}^{2}\equiv E_{\infty}x_{t}x_{t}'=\sum_{n=1}^{\infty} A^{n}GG'(A')^{n}$ [A.1.3]

$S_{\infty}^{2}\equiv\int_{t=0}^{\infty} \exp(f_{x}(\bar{x}(\theta),\theta)t)g(\bar{x}(\theta),\theta)Vg(\bar{x}(\theta),\theta)'\exp(f_{x}(\bar{x}(\theta),\theta)'t)dt$ [A.1.4]

for the case $E_{t}dW_{t}dW_{t}^{'}=Vdt$, provided the linearization is around a stable point so that the infinite matrix series converges. Clearly $S_{\infty}^{2}$ combines the elements of A and G in a complicated formula that makes it hard to disentangle process and noise.

Equation [A.1.3] derives from a recursive formula for the variance matrix at t+1 in terms of the variance matrix at t. Equation [A.1.4] is the continuous time analog and is derived by an analogous argument. From equation [A.1.2], for any time t we have

$S_{t+1}\equiv E(x_{t+1}x_{t+1}^{'})=E\{(Ax_{t}+Ge_{t+1})(Ax_{t}+Ge_{t+1})'\}=AE(x_{t}x_{t}^{'})A'+GG'=AS_{t}A'+GG'$ [A.1.5]

The RHS of [A.1.5] holds because $E\{e_{t+1}e_{t+1}^{'}\}=I,$ and cross terms like $E\{Ge_{t+1}(Ax_{t})'\}=G\{E(e_{t+1}x_{t}^{'})A'\}=0$ because $\{e_{t}\}$ is assumed IID with mean zero and identity variance matrix. Now put $S_{1}=I$ and obtain the sequence

$\begin{matrix} S_{1}=AIA'+GG'=AA'+GG',S_{2}=A(AA'+GG')A'+GG'=A^{2}A'^{2}+AGG'A'+GG', \\ S_{3}=AS_{2}A'+GG'=A(A^{2}A'^{2}+AGG'A'+GG')A'+GG' \\ ... \\ S_{N}=A^{N}A'^{N}+GG'+A(GG')A'+A^{2}(GG')A'^{2}+...+A^{N-1}(GG')A'^{N-1} \end{matrix}$ [A.1.6]

If all the eigenvalues of A are strictly inside the unit circle in the complex plane then

$A^{N}(GG')A'^{N}\to0 as N\to\infty$ [A.1.7]

in the matrix sense and we have equation [A.1.3] above. The recursion in [A.1.6] suggests a computer algorithm for computing $S_{\infty}^{2}$ in cases where A and G are known.

As the ecosystem approaches a critical transition that affects the stability of the equilibrium where we linearized, the eigenvalue of A with largest modulus (or pair of eigenvalues with largest modulus in the complex case) will pass out of the unit circle and S will become infinite. Both of these events occur at the critical point, and both are markers of the critical transition.

However, to have an indicator we need a statistic that shows a signal prior to the critical transition in variables that we can measure. In reality the eigenvalues of $S_{\infty}^{2}$ are almost never measured. Instead we compute statistics for a time series of a variable that is much lower dimension than x. Suppose we are going to compute autocorrelation and variance for a variable y which is lower-dimension than x and related to x by a scaling vector c, so$y_{t}=c'x_{t}$. The long-run variance of y is

$\sigma_{\infty}^{2}=c'S_{\infty}^{2}c$ [A.1.8]

Clearly if there is a critical transition so that S becomes infinite then $\sigma_{\infty}^{2}$also becomes infinite. Note that elements of A and G are combined in [A.1.8], as they are in $S_{\infty}^{2}$. Therefore the trajectory of $\sigma_{\infty}^{2}$as the critical point is approached may or may not be monotonic, depending on details of the elements of A and G.

The long-run autocorrelation of y at lag 1 can be computed from the ordinary least squares regression equation $y_{t+1}=ry_{t}+\varepsilon_{t+1}$:

$\hat{r}_{\infty}=\frac{c'AS_{\infty}^{2}c}{c'S_{\infty}^{2}c}$ [A.1.9]

If the size of the A and S matrices is 2x2 or larger we cannot just cancel out the effect of $S_{\infty}^{2}$ from [A.1.9]. Therefore elements of A and G are combined in the autocorrelation as well as the variance. As in the case of variance, the trajectory of $\hat{r}_{\infty}$may or may not be monotonic as the critical point is approached. In a one-dimensional system, $c'S_{\infty}^{2}c$ terms do cancel in [A.1.9] and autocorrelation is related purely to the eigenvalue of the process (which at equilibrium for one dimension is A). But no important real-world ecosystem is one-dimensional. We may sample a one-dimensional indicator, but we are in fact studying a multi-dimensional ecosystem.

Unfortunately, neither the autocorrelation nor the variance $\sigma_{\infty}^{2}=c'S_{\infty}^{2}c$ depends purely on the leading eigenvalues of $A=f_{x}(\bar{x}(\theta),\theta)$ in the case where the underlying state vector X is multidimensional . This is easy to see by inspection of equation [A.1.9]. Therefore, based upon theoretical considerations, there is no reason to prefer one indicator over the other. However, there could be differences in statistical properties that affect our interpretation of the indicators. In the absence of detailed information about the data generating process [1], the indicators can be estimated by nonparametric regression.

**Literature Cited**

1. Fleming WH (1971) Stochastic control with small noise intensities. Society of Industrial and Applied Mathematics Journal of Control 9: 473-517.

2. Biggs R, Carpenter SR, Brock WA (2009) Turning back from the brink: Detecting an impending regime shift in time to avert it. Proceedings of the National Academy of Sciences of the United States of America 106: 826-831.

3. Kubo R (1966) The fluctuation-dissipation theorem. Reports on Progress in Physics 29: 255-284.
