# Supplementary material for: Early Warnings of Regime Shift When the Ecosystem Structure Is Unknown
Source: PLoS One. 2012 Sep 21;7(9):e45586. doi: 10.1371/journal.pone.0045586 (PMC3448650; doi:10.1371/journal.pone.0045586)
Supplement: Supporting Information S2 — (DOCX) [file pone.0045586.s003.docx]

*Supplementary Information 2. Food Web Model*

Carpenter et al. (2008) present a five-dimensional lake food web model calibrated using long-term data from whole-lake manipulations of food web structure and nutrient input. The model combines a difference equation for piscivore population dynamics with a continuous time model for the other food web components. In order to simplify analysis for the present paper, we created a fully continous-time model by distributing birth and mortality evenly over time. While unrealistic biologically, this assumption simplifies analysis by allowing us to work with a 5-dimensional continuous time system subject to a critical transition caused by the interaction of piscivorous and planktivorous fishes. In both models, transitions from piscivore and planktivore dominance occur as piscivore harvest is gradually increased. Both models address the predatory effects of fish populations on each other and on zooplankton, but do not address the bottom-up effect of prey on predators.

Relevant details of the model are as follows. Parameter values are presented in Carpenter et al. (2008) except where stated otherwise. Dynamics of adult piscivores A follow

$\frac{dA}{dt}=s_{J}J-qEA-m_{A}A+\sigma_{A}\frac{dW_{A}}{dt}$ [A2.1]

where s_J_ is the rate coefficient for maturation of juveniles (J) adults (s_J_ = 0.5 y^-1^); q is catchability; E is angler effort; m_A_ is the mortality rate coefficient for A (m_A_ = 0.5 y^-1^); and σA is the noise magnitude.

Dynamics of juveniles J follow

$\frac{dJ}{dt}=\left(f_{A}+\sigma_{J}\frac{dW_{J}}{dt}\right)A-c_{JA}JA-\frac{c_{JF}vFJ}{h+v+c_{JF}F}-s_{J}J$ [A2.2]

f_A_ is fecundity of adults; c_JA_ is a cannibalism coefficient; c_JF_ is predation on juveniles by planktivores; v is the vulnerability coefficient; h is the hiding coefficient.

Planktivore F dynamics follow

$\frac{dF}{dt}=D_{F}(F_{R}-F)-c_{FA}FA+\sigma_{F}\frac{dW_{F}}{dt}$ [A2.3]

D_F_ is an exchange coefficient between the refuge and open water; F_R_ is the refuge density of planktivores; c_FA_ is consumption coefficient of planktivores by piscivores; σ_F_ is the noise magnitude.

Herbivore (zooplankton) H dynamics follow

$\frac{dH}{dt}=D_{H}(H_{R}-H)+\alpha c_{HP}HP-c_{HF}HF+\sigma_{H}\frac{dW_{H}}{dt}$ [A2.4]

D_H_ is an exchange coefficient between refuge and open water; H_R_ is the refuge density of herbivores; α is the conversion coefficient between phytoplankton and herbivores; c_HP_ is a grazing coefficient; c_HF_ is the predation coefficient for planktivory; σ_H_ is the noise magnitude.

Phytoplankton (P) dynamics follow

$\frac{dP}{dt}=r_{P}L\gamma(I_{0},P)P-mP-c_{PH}HP+\sigma_{P}\frac{dW_{P}}{dt}$ [A2.5]

r_P_ is a growth coefficient; L is phosphorus loading; γ is the growth function described in Carpenter et al. (2008); m is non-grazing mortality; c_PH_ is the grazing coefficient; σ_P_ is the noise magnitude.

The growth function γ imposes self-shading on the phytoplankton which imposes density-dependency on their growth (Carpenter et al. 2008). Because surface irradiance I_0_ is constant in these simulations, the dynamic impact of γ is stabilizing, through density-dependence of P dynamics. Details of the growth function γ are presented in Carpenter et al. (2008) and are not essential for understanding results presented here.

To illustrate the dynamics during a transition in the food web model we present time series from a single realization (Fig. S1). The transition is a fold bifurcation, as in the eutrophication model (Figure S2.1 of Carpenter et al. [[1](#_ENREF_1)]). However, in the food web model the transition takes a long time to occur because piscivore adults are long-lived. Even when piscivore recruitment is shut off by high predation, it takes a long time for the piscivore adults to decline to low density. This provides a useful contrast to the eutrophication model where the critical transition is very fast due to rapid switching of the recycling term at the critical point.

**Literature Cited**

Carpenter, S. R., W. A. Brock, J. J. Cole, J. F. Kitchell, and M. L. Pace. 2008. Leading indicators of trophic cascades. Ecology Letters 11:128-138.

1. Carpenter SR, Brock WA, Cole JJ, Kitchell JF, Pace ML (2008) Leading indicators of trophic cascades. Ecology Letters 11: 128-138.
