# Supplementary material for: Early Warnings of Regime Shift When the Ecosystem Structure Is Unknown
Source: PLoS One. 2012 Sep 21;7(9):e45586. doi: 10.1371/journal.pone.0045586 (PMC3448650; doi:10.1371/journal.pone.0045586)
Supplement: Supporting Information S3 — (DOCX) [file pone.0045586.s004.docx]

*Supporting Information 3: R Function for Computing the Indicators*

# Function to compute nonparametric estimators for time series x

# S.R. Carpenter, December 2011

# Inputs:

# x0 is the regressor

# dx is the first difference of x0

# nx is number of first differences

# DT is time step

# bw is the bandwidth for the kernel

# na is number of a values for computing the kernel

# avec is the mesh for the kernel

Bandi4S2 <- function(x0,dx,nx,DT,bw,na,avec) {

# Set up constants and useful preliminaries

SF <- 1/(bw*sqrt(2*pi)) # scale factor for kernel calculation

x02 <- x0*x0 # second power of x

dx2 <- dx*dx # second power of dx

# Compute matrix of kernel values

Kmat <- matrix(0,nrow=na,ncol=nx)

for(i in 1:(nx)) { # loop over columns (x0 values)

Kmat[,i] <- SF*exp(-0.5*(x0[i]-avec)*(x0[i]-avec)/(bw*bw))

}

# Compute M1, M2, and sum of squares of x0 for each value of a

M1.a <- rep(0,na)

M2.a <- rep(0,na)

mean.a <- rep(0,na)

SS.a <- rep(0,na)

for(i in 1:na) { # loop over rows (a values)

Ksum <- sum(Kmat[i,]) # sum of weights

M1.a[i] <- (1/DT)*sum(Kmat[i,]*dx)/Ksum

M2.a[i] <- (1/DT)*sum(Kmat[i,]*dx2)/Ksum

mean.a[i] <- sum(Kmat[i,]*x0[2:(nx+1)])/Ksum # Buz removes 1/DT on 17 Nov 2011

SS.a[i] <- sum(Kmat[i,]*x02[2:(nx+1)])/Ksum # Buz removes 1/DT on 17 Nov 2011

}

# Compute conditional variance, diffusion and drift functions

S2.x <- SS.a - (mean.a*mean.a) # sum of squares minus squared mean

diff2.x <- M2.a

mu.x <- M1.a

# Return the following functions to the main program:

# mu.x is the drift function

# diff2.x is the diffusion function

# mean.a is the mean function used to compute the conditional variance

# S2.x is the conditional variance function

outlist <- list(mu.x,diff2.x,mean.a,S2.x)

return(outlist)

} # end nonparametric estimates
